# Src Is a Prime Target Inhibited by* Celtis choseniana* Methanol Extract in Its Anti-Inflammatory Action

**DOI:** 10.1155/2018/3909038

**Published:** 2018-03-14

**Authors:** Han Gyung Kim, Subin Choi, Jongsung Lee, Yo Han Hong, Deok Jeong, Keejung Yoon, Deok Hyo Yoon, Gi-Ho Sung, Seungihm Lee, Suntaek Hong, Young-Su Yi, Jong-Hoon Kim, Jae Youl Cho

**Affiliations:** ^1^Department of Genetic Engineering, Sungkyunkwan University, Suwon 16419, Republic of Korea; ^2^Gyeonggi Science High School for the Gifted, Suwon 16297, Republic of Korea; ^3^Institute for Bio-Medical Convergence, International St. Mary's Hospital and College of Medicine, Catholic Kwandong University, Incheon 22711, Republic of Korea; ^4^Department of Biochemistry, Lee Gil Ya Cancer and Diabetes Institute, Gachon University, Incheon 21999, Republic of Korea; ^5^Department of Pharmaceutical Engineering, Cheongju University, Cheongju 28503, Republic of Korea; ^6^College of Veterinary Medicine, Chonbuk National University, Iksan 54596, Republic of Korea

## Abstract

*Celtis choseniana* is the traditional plant used at Korea as a herbal medicine to ameliorate inflammatory responses. Although* Celtis choseniana* has been traditionally used as a herbal medicine at Korea, no systemic research has been conducted on its anti-inflammatory activity. Therefore, the present study explored an anti-inflammatory effect and its underlying molecular mechanism using* Celtis choseniana *methanol extract (Cc-ME) in macrophage-mediated inflammatory responses.* In vitro* anti-inflammatory activity of Cc-ME was evaluated using RAW264.7 cells and peritoneal macrophages stimulated by lipopolysaccharide (LPS), pam3CSK4 (Pam3), or poly(I:C).* In vivo* anti-inflammatory activity of Cc-ME was investigated using acute inflammatory disease mouse models, such as LPS-induced peritonitis and HCl/EtOH-induced gastritis. The molecular mechanism of Cc-ME-mediated anti-inflammatory activity was examined by Western blot analysis and immunoprecipitation using whole cell and nuclear fraction prepared from the LPS-stimulated RAW264.7 cells and HEK293 cells. Cc-ME inhibited NO production and mRNA expression of inducible nitric oxide synthase (iNOS), cyclooxygenase (COX-2), and tumor necrosis factor-alpha (TNF-*α*) in the RAW264.7 cells and peritoneal macrophages induced by LPS, pam3, or poly(I:C) without cytotoxicity. High-performance liquid chromatography (HPLC) analysis showed that Cc-ME contained anti-inflammatory flavonoids quercetin, luteolin, and kaempferol. Among those, the content of luteolin, which showed an inhibitory effect on NO production, was highest. Cc-ME suppressed the NF-*κ*B signaling pathway by targeting Src and interrupting molecular interactions between Src and p85, its downstream kinase. Moreover, Cc-ME ameliorated the morphological finding of peritonitis and gastritis in the mouse disease models. Therefore, these results suggest that Cc-ME exerted* in vitro* and* in vivo* anti-inflammatory activity in LPS-stimulated macrophages and mouse models of acute inflammatory diseases. This anti-inflammatory activity of Cc-ME was dominantly mediated by targeting Src in NF-*κ*B signaling pathway during macrophage-mediated inflammatory responses.

## 1. Introduction

Inflammation is the defensive process that protects a human body from infection by various pathogens, including bacteria, viruses, fungi, and protozoans, and is mainly induced by innate immune cells, including phagocytic macrophages, neutrophils, and dendritic cells [1–3]. Innate immune cells recognize the pathogen-associated molecular patterns of invading pathogens through special pattern recognition receptors (PRRs), including toll-like receptors (TLRs). They then initiate inflammatory responses by activating several inflammatory signaling pathways, including nuclear factor-kappa B (NF-*κ*B), activated protein-1 (AP-1), and interferon-regulatory factors (IRFs), along with the intracellular signaling molecules in these pathways [4–7]. Lipopolysaccharide (LPS) is one of the most powerful agonists for activating inflammatory responses in macrophages that bind to its molecular receptor, TLR4, which transduces inflammatory signal cascades via two major adaptor molecules, toll/interleukin-1 receptor-domain-containing, adapter-inducing interferon-*β* (TRIF), and myeloid differentiation response gene 88 (MyD88) [8]. Sequential activation of these proteins induces the expression of proinflammatory cytokines and genes, including tumor necrosis factor-alpha (TNF-*α*), interleukin-1*β* (IL-1*β*), IL-6, inducible nitric oxide synthase (iNOS), and cyclooxygenase-2 (COX-2), and the production of inflammatory mediators, including prostaglandin E_2_ (PGE_2_) and nitric oxide (NO) [4–7]. Increased expression and production of these genes and mediators promote inflammatory responses and induce phagocytosis activity among innate immune cells [9, 10].

Although inflammation plays pivotal roles in protecting a human body from invading pathogens, it also induces heat, pain, redness, swelling, and loss of function. Prolonged inflammation (known as chronic inflammation) causes damage at the vascular, cellular, and lymphatic levels, causing serious diseases, such as inflammatory/autoimmune disorders, atherosclerosis, type I diabetes mellitus, and even cancers [11, 12]. Therefore, research to identify and validate anti-inflammatory therapeutics is crucial to preventing and treating those diseases. For such substances to be used as drugs, they must be shown to have anti-inflammatory effects and be safe for use. A variety of plants have been traditionally used as medicinal herbs for a long time because of their therapeutic effects and safety.

*Celtis choseniana*, in the Ulmaceae family, is an endemic species found in every part of Korea [13, 14].* Celtis *genus was traditionally used at Eastern Asia, including Korea, China, and Japan, as medicinal plants, and their leaves and barks were used to treat pain, boils, lung abscess, gastric disease, irregular menstruation, and lumbago [15–17]. In spite of the fact that this plant has been used for treating inflammation-related diseases, no previous systemic research of its therapeutic effects on inflammatory responses or diseases has been reported. Thus, in the present study, we first investigated and reported an anti-inflammatory activity of* Celtis choseniana* and its underlying molecular mechanism in macrophage-mediated inflammatory responses.

## 2. Materials and Methods

### 2.1. Materials

An 80% methanol extract of the leaves of* Celtis choseniana *(Cc-ME) was obtained from Korea Plant Extract Bank (Cheongju, Korea). N(G)-Nitro-L-arginine methyl ester (L-NAME), (3-4,5-dimethylthiazol-2-yl)-2,5-diphenyltetrazolium bromide (MTT), sodium dodecyl sulfate (SDS), prednisolone (Pred), ranitidine, luteolin, kaempferol, quercetin, pam3CSK4 (Pam3), poly(I:C), polyethylene imidazole (PEI), and lipopolysaccharide (LPS) were purchased from Sigma Chemical Co. (St. Louis, MO, USA). F4/80 (F4/80-FITC^,^ a marker for macrophages), B220 (B220-PE, a maker for B cells), and CD3 (CD3-APC, a marker for T cells) were purchased from BD Biosciences (San Jose, CA, USA) and ThermoFisher Scientific (Waltham, MA, USA). Fetal bovine serum (FBS), phosphate buffered saline (PBS), TRIzol reagent, Dulbecco's Modified Eagle's medium (DMEM), and Roswell Park Memorial Institute (RPMI) 1640 were purchased from GIBCO (Grand Island, NY, USA). RAW264.7 cells (ATCC number TIB-71) and HEK293 cells (ATCC number CRL-1573) were purchased from the* American* Type Culture Collection (ATCC) (Rockville, MD, USA). Antibodies specific for phosphorylated and total forms of p65, p50, Lamin A/C, IKK*α*/*β*, I*κ*B*α*, Src, p85/PI3K, AKT, and *β*-actin were purchased from Cell Signaling Technology (Beverly, MA, USA).

### 2.2. Animals

ICR and C57BL/6 mice (male, 6–8 weeks old) were purchased from Daehan Biolink (Osong, Korea). The mice were maintained in the plastic cages at a customary condition. Water and pelleted diet (Samyang, Daejeon, Korea) were provided ad libitum. All studies were conducted according to the guideline of the Institutional Animal Care and Use Committee at Sungkyunkwan University (Suwon, Korea; approval ID: SKKUBBI 12-6-1).

### 2.3. Cell Culture and Cc-ME Treatment

RAW264.7 and HEK293 cell lines were maintained in RPMI 1640 and DMEM, respectively, supplemented with glutamine, 10% heat-inactivated FBS, penicillin, and streptomycin. The cells were cultured at 37°C with 5% CO_2_ supplementation in a humidified incubator. For* in vitro* experiments, Cc-ME stock solution (200 mg/ml) was prepared in dimethyl sulfoxide (DMSO) and diluted to 0–100 *μ*g/ml using cell culture media in use. For* in vivo* conditions, Cc-ME was suspended in 0.5% sodium carboxymethyl cellulose (sodium CMC) to prepare effective doses (50 and 200 mg/kg), which were previously proposed for pharmacological tests with medicinal plant-derived methanol or ethanol extracts [18–20].

### 2.4. Preparation of Peritoneal Macrophages

Peritoneal exudates were procured in C57BL/6 male mice. 4% sterile thioglycollate broth (1.0 ml, Difco Laboratories, Detroit, MI) was intraperitoneally injected for 4 d [21, 22]. Exudates were washed three times using RPMI 1640 media containing 10% FBS, and peritoneal macrophages were plated in culture plates for each experiment.

### 2.5. NO Production Assay

RAW264.7 cells and peritoneal macrophages (5 × 10^6^ cells/ml) pretreated with either Cc-ME (0–100 *μ*g/ml) or standard compounds (L-NAME or luteolin) for 30 min were treated with inflammatory stimuli [LPS (1 *μ*g/ml), Pam3 (10 *μ*g/ml), or poly(I:C) (200 *μ*g/ml)] for 24 h. Suppressive effect of Cc-ME on NO production was examined by measuring NO level in the cell culture medium using Griess reagents [23].

### 2.6. Cell Viability Assay

RAW264.7 cells or peritoneal macrophages (5 × 10^6^ cells/ml) were treated with Cc-ME (0–100 *μ*g/ml) for 24 h, and cytotoxicity of Cc-ME for the cells was examined by determining cell viability using the MTT assay [8, 24]. Briefly, 10 *μ*l of MTT solution (10 mg/ml in PBS, pH 7.4) was added to cell culture media, and, 4 h later, 100 *μ*l of 15% SDS solution was mixed to solubilize formazan crystals [25]. Absorbance was determined at 570 nm by a Spectramax 250 microplate reader.

### 2.7. HPLC Analysis

The components of* Celtis choseniana* were analyzed by high-performance liquid chromatography (HPLC) according to previous conditions [26]. Quercetin, luteolin, and kaempferol were used as standard compounds for HPLC. The conditions of the HPLC analysis are described in Table 1.

### 2.8. Analysis of mRNA Expression by Semiquantitative and Quantitative Reverse Transcription-Polymerase Chain Reaction (RT-PCR)

RAW264.7 cells pretreated with Cc-ME (0, 50, and 100 *μ*g/ml) for 30 min were treated with LPS (1 *μ*g/ml) for 6 h. Total RNA was extracted by TRI Reagent® solution following the manufacturer's instructions and stored at −70°C until use. Quantification of mRNA expression levels of iNOS, TNF-*α*, and COX-2 was performed by semiquantitative and quantitative real-time RT-PCR [27, 28]. Primer sequences for RT-PCR are listed in Table 2.

### 2.9. Preparation of Total Cell and Nuclear Lysates

RAW264.7 cells (1 × 10^6^ cells/ml) were washed with ice-cold PBS including 1 mM sodium orthovanadate and lysed with ice-cold lysis buffer (20 mM Tris-HCl, pH 7.4, 2 mM EDTA, 2 mM EGTA, 50 mM glycerol phosphate, 1 mM DTT, 2 *μ*g/ml aprotinin, 2 *μ*g/ml leupeptin, 1 *μ*g/ml pepstatin, 50 *μ*M PMSF, 1 mM benzamide, 2% Triton X-100, 10% glycerol, 0.1 mM sodium vanadate, 1.6 mM pervanadate, and 20 mM NaF). Total cell lysates were clarified by centrifugation at 12,000 rpm for 5 min at 4°C and stored at −20°C until used. Nuclear lysates were prepared, as previously described [29, 30]. Briefly, the cells were washed with 1x PBS and centrifuged at 12,000 rpm for 5 min. Supernatant was discarded, and cell pellets were lysed in 400 *μ*l of ice-cold lysis buffer (10 mM HEPES pH 7.8, 10 mM KCl, 2 mM MgCl_2_, 0.1 mM EDTA, 1 mM DTT, 0.1 mM PMSF, 2 *μ*g/ml leupeptin, and 2 *μ*g/ml aprotinin). Next, 25 *μ*l of 10% NP-40 was added to the lysates, and the lysates were vigorously mixed by vortexing. The lysates were then centrifuged at 14,000 rpm for 0.5 min at 4°C, and the supernatant was removed. Pellets were washed with 400 *μ*l of lysis buffer and 50 *μ*l of extraction buffer (10 mM HEPES pH 7.8, 50 mM KCl, 400 mM NaCl, 0.1 mM EDTA, 1 mM DTT, 0.1 mM PMSF, 2 *μ*g/ml leupeptin, 2 *μ*g/ml aprotinin, and 10% glycerol). The solution was stored at 4°C for 20 min and centrifuged at 14000 rpm at 4°C for 5 min. Supernatant was transferred to fresh tube and stored at −80°C until use.

### 2.10. Western Blot Analysis and Immunoprecipitation

Proteins in total cell lysates and nuclear lysates were electrophoresed on SDS-polyacrylamide gels (30% acrylamide, Tris-base, 10% SDS, 10% APS, TEMED, pH 8.8) using running buffer (Tris-base, glycine, 10% SDS). The proteins were transferred by electroblotting to a polyvinylidene difluoride membrane using transfer buffer (Tris-base, glycine, 10% SDS, methanol). The membranes were blocked using 5% bovine serum albumin (BSA) in PBS at room temperature for 1 h, followed by the incubation with specific primary antibodies for 1 h at room temperature. After primary antibody incubation, the membranes were washed with 0.1% TBST (Tris-base, NaCl, 0.1% Tween 20, pH 7.6) 3 times for 10 min each, incubated with HRP-linked secondary antibodies with 3% BSA for 1 h at room temperature and washed with 0.1% TBST 3 times for 10 min each. Phosphorylated and total forms of p65, p50, Src, p85, AKT, IKK*α*, I*κ*B*α*, *β*-actin, and Lamin A/C were visualized using an ECL reagent [31]. For immunoprecipitation, cell lysates containing equal amounts of proteins were precleared with 10 *μ*l of protein A-coupled Sepharose beads (50% v/v) for 3 h at 4°C. Precleared samples were incubated with the antibodies specific for Src and p85 overnight at 4°C. Immunocomplexes were mixed with 10 *μ*l of protein A-coupled Sepharose beads (50% v/v) and incubated for 4 h at 4°C. After removing supernatant, the beads were washed with PBS 5 times, followed by boiling for 2 min in protein sample buffer for immunoprecipitation. Immunoprecipitates were analyzed by Western blotting to investigate phosphorylated or total levels of transcription factors and visualized using an ECL reagent [32].

### 2.11. LPS-Induced Peritonitis in Mice

To generate peritoneal exudates, C57BL/6 mice (*n* = 10/group) were intraperitoneally injected with 1.0 ml of sterile 4% thioglycollate broth, as previously described [33, 34]. Right after, Cc-ME (0, 50, and 200 mg/kg) suspended in 0.5% sodium CMC was orally administered by gavage once per day for 5 days. Acute peritonitis was induced by intraperitoneal injection of 1.0 ml (10 mg/kg) of LPS (*E. coli* 0111:B4) on day 4. On day 5, peritoneal fluid was collected by peritoneal lavage using sterile PBS. The effect of Cc-ME on peritonitis was examined by counting total leukocytes in a Neubauer chamber after staining with Turk solution.

### 2.12. Flowcytometry Analysis

To analyze the population of immune cells in peritoneal exudates, flowcytometric analyses were carried out. Briefly, the cells from exudates were washed with FACS buffer (phosphate buffer saline containing 2% fetal calf serum and 0.1% sodium azide) and then incubated in 50 mL FACS buffer containing 10% rabbit serum for 10 min on ice. The cells were incubated with the indicated fluorochrome-conjugated antibodies specific for F4/80 (F4/80-FITC, a marker for macrophages), B220 (B220-PE, a maker for B cells), and CD3 (CD3-APC, a marker for T cells) for 45 min on ice and washed three times with ice-cold FACS buffer. The stained cells were analyzed by a FACSCalibur instrument (Becton Dickinson, Mountain View, CA, USA).

### 2.13. EtOH/HCl-Induced Gastritis in Mice

Acute stomach inflammation was induced in ICR mice using EtOH/HCl according to the method, described previously [35, 36]. After anaesthetizing and sacrificing the mice using urethane 1 h after the administration of necrotizing agents, the stomachs of the mice were excised and rinsed under running tap water. After opening the stomachs along the greater curvature and spreading them out on a board, the area (mm^2^) of mucosal erosive lesions was measured using a pixel counter by a person blinded to the treatment condition.

### 2.14. Statistical Analysis

**Figure 1:**
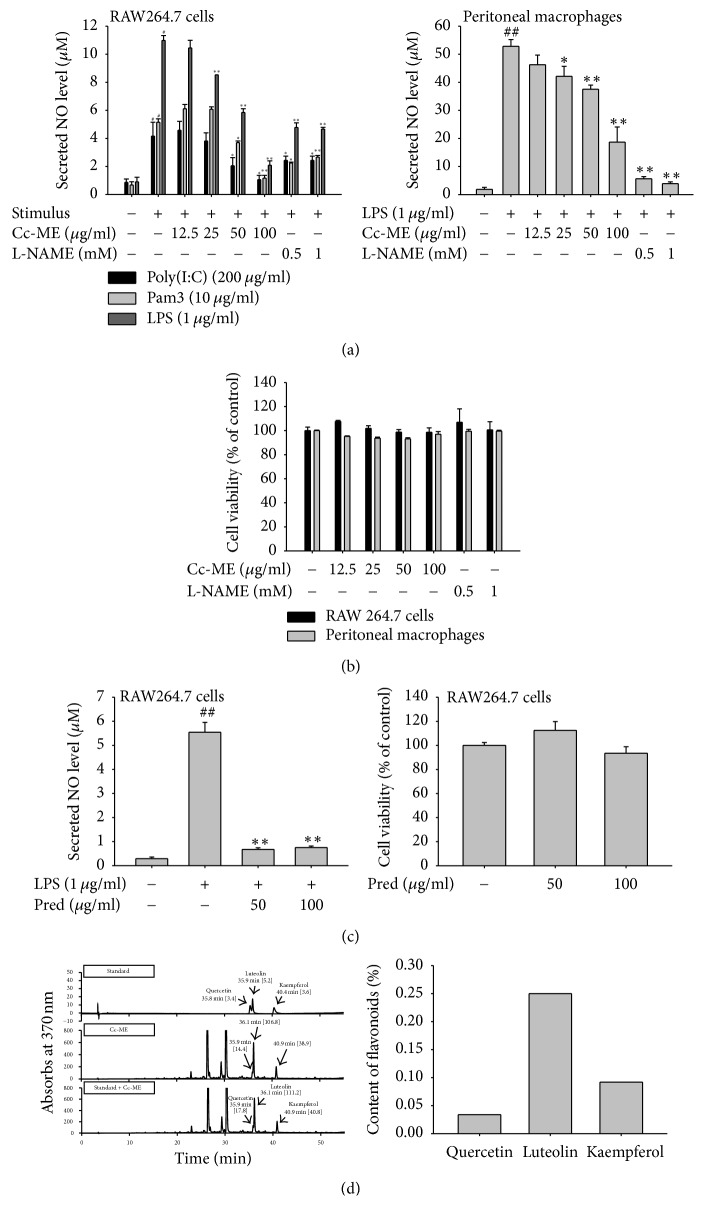
*Effects of Cc-ME on NO production and cytotoxicity in macrophages and HPLC analysis of Cc-ME components.* (a) RAW264.7 cells (left panel) or peritoneal macrophages (right panel) pretreated with the indicated doses of Cc-ME or L-NAME were treated with LPS (1 *μ*g/ml), pam3 (10 *μ*g/ml), or poly(I:C) (200 *μ*g/ml) for 24 h, and NO production level in the cells was determined by the Griess assay. (b) RAW264.7 cells or peritoneal macrophages were treated with the indicated doses of Cc-ME or L-NAME for 24 h, and the viabilities of the cells were determined by the MTT assay. (c) The NO inhibitory activity (left panel) of prednisolone (Pred) and its cytotoxicity (right panel) were examined by NO assay and MTT assay. (d) The phytochemical profile (left panel) of Cc-ME was analyzed by HPLC using standard compounds (quercetin, luteolin, and kaempferol) in the conditions described in Table 1, and the contents of the flavonoids (right panel) were calculated from the standard curves of the standard compounds. The data presented in (a), (b), and (c) are expressed as the means ± SD of experiments that were performed with six. ^#^*P* < 0.05 and ^##^*P* < 0.01 compared to normal group; ^*∗*^*P* < 0.05 and ^*∗∗*^*P* < 0.01 compared to control group.

**Figure 2:**
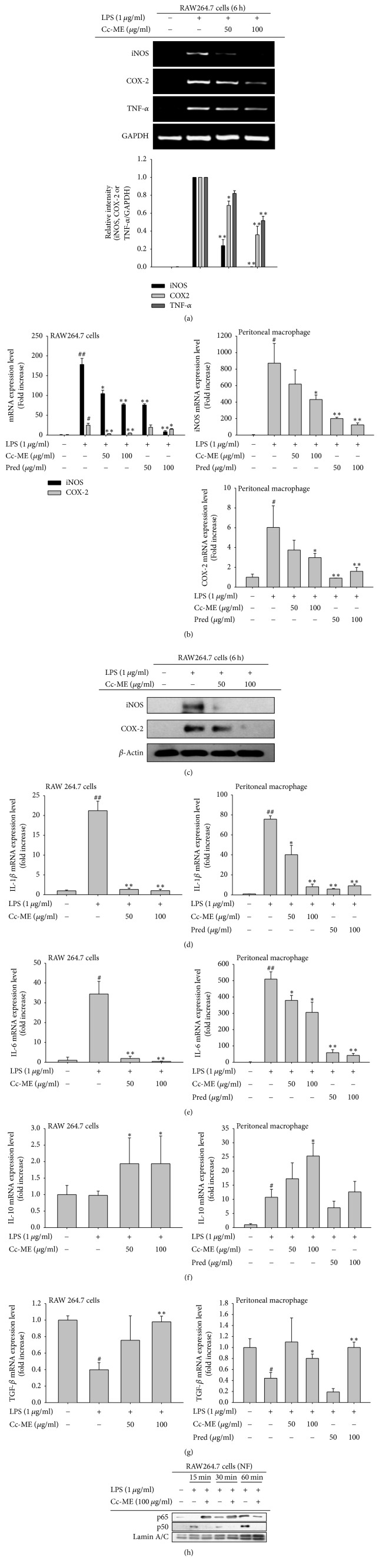
*Effects of Cc-ME on mRNA and protein expression of inflammatory genes.* RAW264.7 cells or peritoneal macrophages pretreated with the indicated doses of Cc-ME or standard drug (Pred) for 30 min were treated with LPS (1 *μ*g/ml) for 6 h. (a, b, d, e, f, and g) mRNA and protein expression levels of iNOS, COX-2, TGF-*β*, IL-10, IL-6, IL-1*β*, and TNF-*α* were determined by semiquantitative RT-PCR (a) or by quantitative real-time PCR (b, d, e, f, and g). (c and h) RAW264.7 cells pretreated with Cc-ME (50 and 100 *μ*g/ml) for 30 min were treated with LPS (1 *μ*g/ml) for the indicated time, and total levels of iNOS and COX-2 in whole cell lysates or p65 and p50 in nuclear fractions were examined by Western blot analysis. *β*-Actin and Lamin A/C were used as internal controls for whole cell lysate and nuclear fractions, respectively. The data presented in (a), (c), and (h) are a representative of three experiments. The data presented in (b), (d), (e), (f), and (g) are expressed as the means ± SD of experiments that were performed with six samples. Relative intensity (bottom panel of (a)) were expressed as means ± SD, calculated with data observed by three experiments using the DNR Bioimaging system. NF: nuclear fraction. ^#^*P* < 0.05 and ^##^*P* < 0.01 compared to normal group, and ^*∗*^*P* < 0.05 and ^*∗∗*^*P* < 0.01 compared to control group.

**Figure 3:**
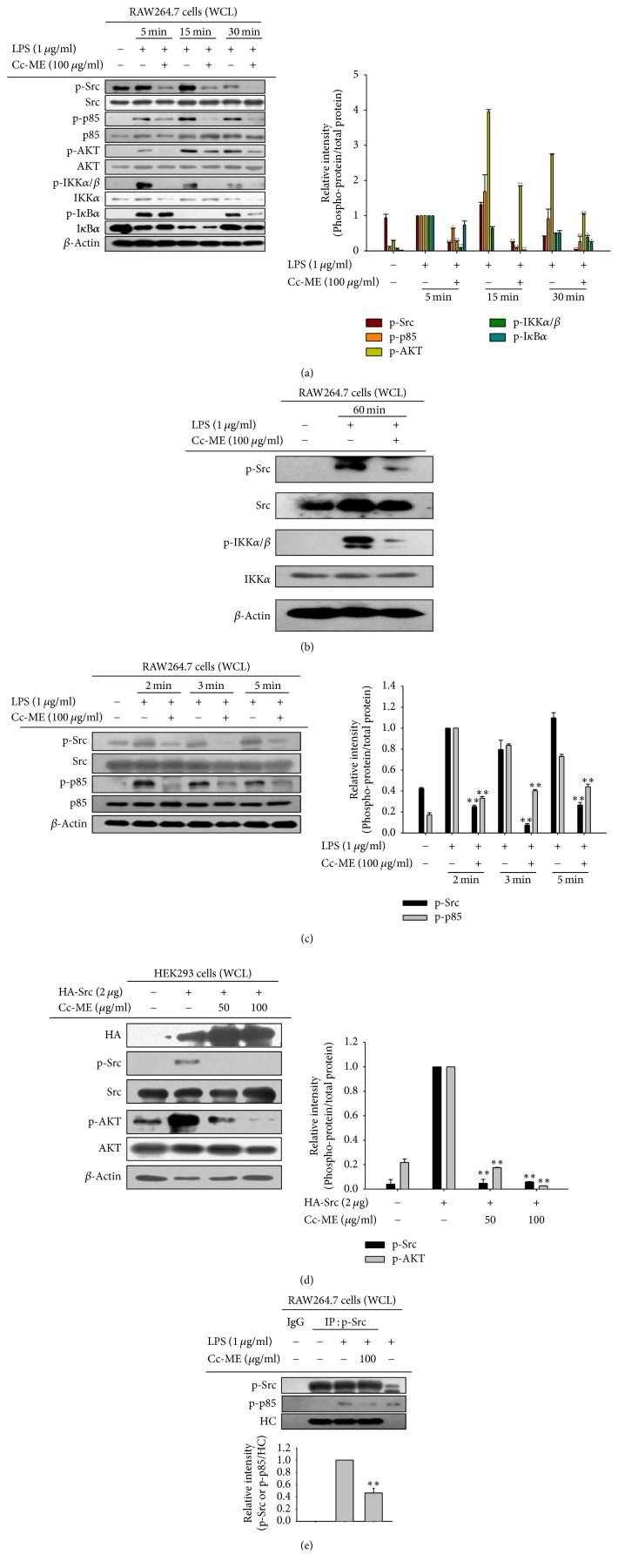
*The effect of Cc-ME on activation of the NF-κB signaling pathway.* (a, b, and c) RAW264.7 cells pretreated with Cc-ME (100 *μ*g/ml) for 30 min were treated with LPS (1 *μ*g/ml) for the indicated time, and phosphorylated and total forms of Src, p85, AKT, IKK*α*/*β*, I*κ*B*α*, and *β*-actin were examined by Western blot analysis using their specific antibodies. (d) HEK293 cells were transfected with HA-Src for 48 h, and phosphorylated and total forms of Src, p85, AKT, IKK*α*/*β*, I*κ*B*α*, and *β*-actin were examined by Western blot analysis using their specific antibodies. (e) RAW264.7 cells pretreated with Cc-ME (100 *μ*g/ml) for 30 min were treated with LPS (1 *μ*g/ml) for 5 min. Phosphorylated Src was immunoprecipitated in the total cell lysates of the cells, and phosphorylated p85 was examined by Western blot analysis using its specific antibody. The data presented in (a), (b), (c), (d), and (e) are a representative of three experiments. Relative intensity (right panels of (a), (c), and (d), and bottom panel of (e)) was expressed as means ± SD, calculated with data observed by three experiments using the DNR Bioimaging system. WCL: whole cell lysate. HC: heavy chain. ^*∗*^*P* < 0.05 and ^*∗∗*^*P* < 0.01 compared to control group.

**Figure 4:**
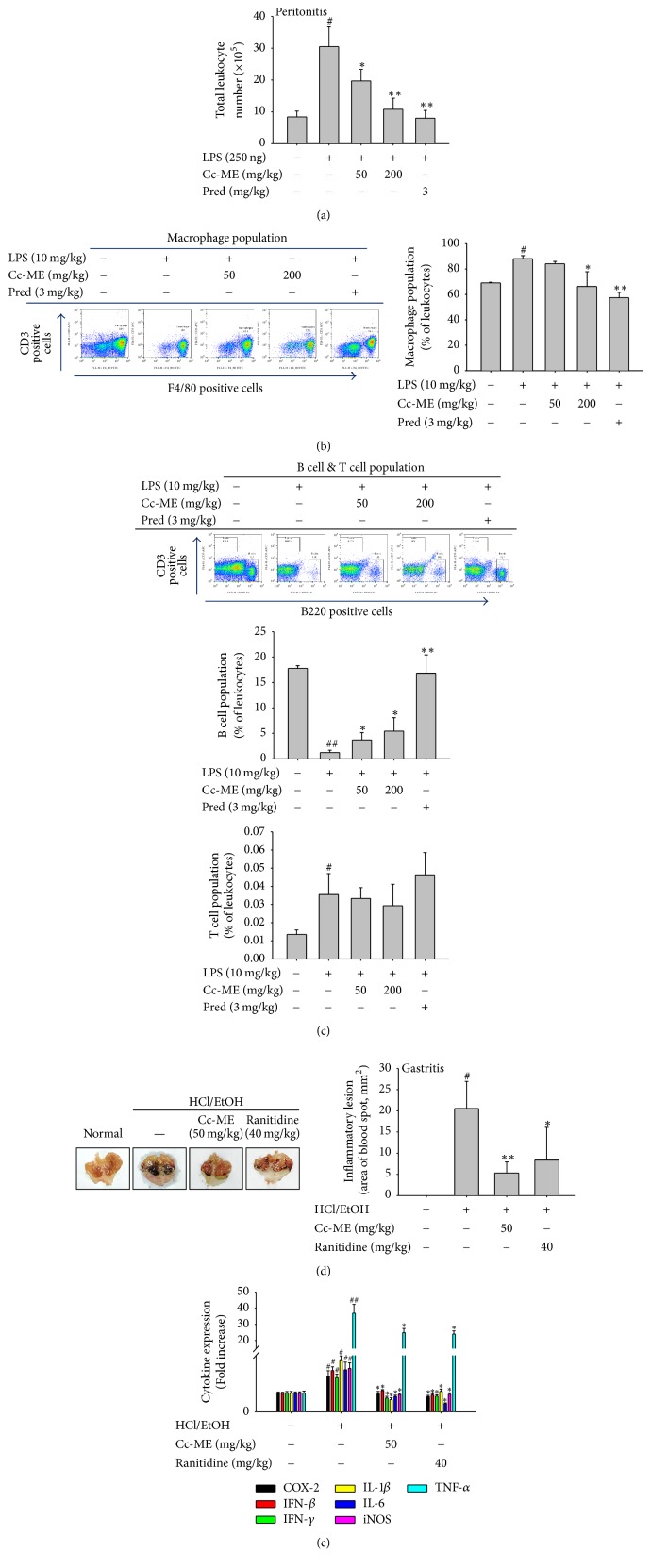
*In vivo effects of Cc-ME on inflammatory morphological finding in LPS-induced peritonitis and HCl/EtOH-induced gastritis in mice*. (a, b, and c) After sterile thioglycollate broth (4%) was injected to mice, Cc-ME (0, 50, and 200 mg/kg) or prednisolone (Pred, 3 mg/kg) was also orally administered every day for 5 days. At day 4, LPS was intraperitoneally injected to these mice. (a) At day 5, peritoneal exudates were collected using sterile PBS, and total leukocytes were counted using a Neubauer chamber after staining with Turk solution. (b and c) The numbers of macrophages (b), B cells ((c) middle panel), or T cells ((c) lower panel) in exudates were analyzed by flowcytometry after staining with F4/80 for macrophages, B220 for B cells, or CD3 for T cells. (d and e) Mice were orally given Cc-ME (0 and 50 mg/kg) or ranitidine (40 mg/kg) every day for 3 days before oral administration of HCl/EtOH. 1 h after oral administration of HCl/EtOH, stomachs of the mice were excised, and the gastritis lesions in the stomachs were photographed ((d) left panel) and measured using an ImageJ software program ((d) right panel). (e) mRNA expression levels of iNOS, COX-2, IFN-*β*, IFN-*γ*, IL-1*β*, IL-6, and TNF-*α* from stomach tissues of mice treated with HCl/EtOH were determined by quantitative real-time PCR. The data presented in (a) and (b) are expressed as the means ± SD of experiments that were performed with seven (b) or ten (a) mice per group. Pred: prednisolone. ^#^*P* < 0.05 and ^##^*P* < 0.01 compared to normal group, and ^*∗*^*P* < 0.05 and ^*∗∗*^*P* < 0.01 compared to control group.

All of the data presented in this paper are expressed as the means ± SD of experiments that were performed with six (Figures 1(a), 1(b), and 1(c)) or three (Figures 1(d), 2, 3, and 4(c)) samples for the* in vitro *experiments and seven (gastritis, Figure 4(b)) or ten (peritonitis, Figure 4(a)) mice for the* in vivo* tests. For the statistical comparisons, the results were analyzed using either ANOVA/Scheffe's post hoc test or the Kruskal-Wallis/Mann–Whitney test. A *P* value < 0.05 was considered to be a statistically significant difference. All of the statistical tests were carried out using the computer program SPSS (SPSS Inc., Chicago, IL). Similar experimental data were also observed using an additional independent set of* in vitro* and* in vivo* experiments that was conducted using the same numbers of samples or mice.

## 3. Results

### 3.1. *In Vitro* Anti-Inflammatory Effect of Cc-ME

To examine an anti-inflammatory activity of Cc-ME in macrophages, we first determined the level of NO production, a critical event in inflammatory reaction, in the Cc-ME-treated RAW264.7 cells and peritoneal macrophages treated with TLR ligands such as LPS (ligand of TLR4) derived from Gram (−) bacteria, Pam3CSK (ligand of TLR1/2) derived from Gram (+) bacterial, or poly(I:C) (ligand of TLR3) derived from virus. NO production was significantly suppressed by Cc-ME treatment in the LPS-treated RAW264.7 cells in a dose-dependent manner (Figure 1(a), left panel), LPS-stimulated peritoneal macrophages (Figure 1(a), right panel), pam3-treated RAW264.7 cells (Figure 1(a), left panel), and poly(I:C)-treated RAW264.7 cells (Figure 1(a), left panel). L-NAME (0.5 and 1 mM) and prednisolone (Pred, 50 and 100 *μ*g/ml), standard anti-inflammatory compounds, also displayed an inhibitory pattern under the same NO production condition in the RAW264.7 cells and peritoneal macrophages stimulated with TLR ligands (Figures 1(a) and 1(c)). To examine the cytotoxicity of Cc-ME on macrophages, we treated RAW264.7 cells and peritoneal macrophages with escalating doses of Cc-ME and determined cell viability. The viabilities of RAW264.7 cells or peritoneal macrophages were not changed at any dose (Figure 1(b)). L-NAME and Pred also did not affect the cell viability of either type of cells as well (Figures 1(b) and 1(c) left panel).

To identify anti-inflammatory components in Cc-ME, we subjected Cc-ME to HPLC analysis using the standard anti-inflammatory flavonoids quercetin, luteolin, and kaempferol [37, 38]. HPLC analysis demonstrated that Cc-ME contains quercetin, luteolin, and kaempferol (Figure 1(c), left panel), and the content of luteolin was the highest among them (Figure 1(c), right panel). Because Cc-ME contained high levels of luteolin, we also tested its anti-inflammatory effects in RAW264.7 cells and peritoneal macrophages treated with LPS. Luteolin effectively suppressed NO production in both RAW264.7 cells and peritoneal macrophages in a dose-dependent manner (data not shown), as reported previously [39].

### 3.2. Suppressive Effect of Cc-ME on Inflammatory Gene Expression at a Transcriptional Level

To examine the effects of Cc-ME on inflammatory gene expression at a transcriptional level in macrophages, we stimulated RAW264.7 cells pretreated with escalating doses of Cc-ME with LPS and determined mRNA and protein expression levels of inflammatory genes and level of transcription factors translocated into the nucleus using both semiquantitative and real-time PCR with total RNA and Western blot analysis with whole cell lysates and nuclear extracts (NF). Semiquantitative RT-PCR results showed that Cc-ME effectively suppressed mRNA expression of inflammatory genes (iNOS, COX-2, and TNF-*α*) in the LPS-stimulated RAW264.7 cells in a dose-dependent manner (Figure 2(a)). Similarly, quantitative real-time RT-PCR results revealed that Cc-ME as well as Pred significantly decreased mRNA expression of iNOS and COX-2 in the LPS-stimulated RAW264.7 cells in a dose-dependent manner (Figure 2(b) left panel). Similar inhibitory patterns of iNOS and COX-2 gene expression by Cc-ME were also observed in activated peritoneal macrophages. Thus, 100 *μ*g/ml of Cc-ME reduced the mRNA expression of these genes in peritoneal macrophages under LPS-treated conditions up to 56% and 53% (Figure 2(b) right upper and lower panels). Furthermore, the protein levels of iNOS and COX-2 were also reduced by Cc-ME (Figure 2(c)). In addition, to check the effect on the expression of proinflammatory (IL-1*β*, IL-6, and TNF-*α*) and anti-inflammatory (IL-10 and TGF-*β*) cytokines, the mRNA levels of IL-1*β*, IL-6, and TNF-*α* as well as IL-10 and TGF-*β* were determined by real-time PCR from both RAW264.7 cells and peritoneal macrophages. As Figures 2(d) and 2(e) show, Cc-ME strongly or significantly reduced the expression of IL-1*β* and IL-6, while the expression level of TNF-*α* in LPS-treated peritoneal macrophages was only downregulated by Cc-ME (100 *μ*g/ml) up to 25% (data not shown). Interestingly, however, the expression levels of anti-inflammatory cytokines (IL-10 and TGF-*β*) from both RAW264.7 cells and peritoneal macrophages were increased up to 2-fold or 2.5-fold compared to LPS alone or fully recovered up to their normal levels in Cc-ME-treated groups (Figures 2(f) and 2(g)). To examine the effects of Cc-ME on the activity of NF-*κ*B in LPS-stimulated RAW264.7 cells, RAW264.7 cells pretreated with 100 *μ*g/ml of Cc-ME were stimulated with LPS, and nuclear translocation of NF-*κ*B (p65 and p50) was determined. As shown in Figure 2(h), Cc-ME effectively inhibited nuclear translocation of both p65 and p50 in RAW264.7 cells 60 min after LPS stimulation. Meanwhile, since Cc-ME failed to suppress the activation of AP-1 triggered by TLR4 adaptor molecules (MyD88 and TRIF) (data not shown), we further focused on NF-*κ*B activation signaling events.

### 3.3. Suppressive Effect of Cc-ME on Upstream Signaling for NF-*κ*B Activation

To examine the effects of Cc-ME on the activity of upstream molecules for the NF-*κ*B signaling pathway, we determined the phosphorylation of those upstream molecules in LPS-treated RAW264.7 cells after Cc-ME treatment using Western blot analysis and immunoprecipitation. Since I*κ*B*α*, IKK*α*/*β*, AKT, p85, and Src are known as upstream regulators of NF-*κ*B translocation into the nucleus, the activation of these molecules was assessed by determining their phosphorylation state using their phospho-specific antibodies as reported previously [40, 41]. Phosphorylation of I*κ*B*α* and IKK*α*/*β* was inhibited by Cc-ME from 30 to 60 min, and phosphorylation of AKT and p85 was suppressed by Cc-ME from 5 to 60 min in the LPS-treated RAW264.7 cells (Figures 3(a) and 3(b)). Phosphorylation of Src was inhibited by Cc-ME from 5 to 30 min or 60 min in the LPS-simulated RAW264.7 cells (Figures 3(a) and 3(b)). Next, we determined the effects of Cc-ME on phosphorylation of Src and p85 at early time points. Phosphorylation of both Src and p85 at 2, 3, and 5 min was suppressed by Cc-ME in the LPS-treated RAW264.7 cells (Figure 3(c)). The effect of Cc-ME on upstream molecules of the NF-*κ*B signaling pathway was further examined in HEK293 cells transfected with Src. Cc-ME dramatically suppressed the phosphorylation of Src and AKT induced by Src transfection in HEK293 cells in a dose-dependent manner (Figure 3(d)). Finally, the effect of Cc-ME on the interaction between Src and p85 was examined in LPS-treated RAW264.7 cells. Immunoprecipitation and Western blotting results clearly showed that the interaction between Src and p85 was inhibited by Cc-ME in LPS-treated RAW264.7 cells (Figure 3(e)).

### 3.4. *In Vivo* Anti-Inflammatory Effect of Cc-ME

To check whether the pharmacological efficacy of Cc-ME can be proved by oral administration* in vivo* and whether Cc-ME can reduce both LPS-dependent and LPS–independent inflammatory disorders, we employed LPS-triggered peritonitis and HCl/EtOH-induced gastritis conditions, as reported previously [33, 35]. Cc-ME significantly reduced the number of leukocytes in the peritoneal exudates of the peritonitis mice in a dose-dependent manner (Figure 4(a)). A standard anti-inflammatory agent, prednisolone (Pred), also reduced the number of leukocytes in the peritoneal exudates of the peritonitis mice (Figure 4(a)). By flowcytomeric analysis, which cell population is suppressed by Cc-ME was examined. As Figures 4(b) and 4(c) show, it was revealed that Cc-ME can reduce the numbers of macrophages, while there was no reduction of T cells or little increase in B cell population by this extract (Figures 4(b) and 4(c)). Moreover, Cc-ME ameliorated the morphological finding of gastritis (Figure 4(d), left panel) and decreased the area of blood spots in the stomach inflammatory lesions (Figure 4(d), right panel). In addition, similar to phenotype of Cc-ME in gastritis model, increased mRNA levels of inflammatory genes (COX-2, IFN-*β*, IFN-*γ*, IL-1*β*, IL-6, iNOS, and TNF-*α*) were also significantly suppressed in both Cc-ME- and ranitidine-treated groups (Figure 4(e)).

## 4. Discussion

In the present study, we explored an anti-inflammatory activity of Cc-ME* in vitro* using macrophages and* in vivo* using acute inflammatory disease models in mice. Moreover, we examined the underlying molecular mechanism of the anti-inflammatory activity of Cc-ME in macrophages. NO is one of the major inflammatory mediators generated in macrophages during inflammatory responses; therefore, we first examined* in vitro* anti-inflammatory activity of Cc-ME by determining NO production levels in macrophages. Because TLR2, TLR3, and TLR4 are all critical PRRs that induce inflammatory responses in macrophages, an anti-inflammatory activity of Cc-ME was investigated in macrophages in which inflammatory responses are induced by specific ligands of TLR2, TLR3, and TLR4: pam3, poly(I:C), and LPS, respectively. Cc-ME clearly suppressed activity in the production of NO induced by all three ligands without generating cytotoxicity (Figures 1(a) and 1(b)), indicating that Cc-ME's* in vitro* anti-inflammatory activity is due to its ability to decrease NO production level in macrophages. Likewise, this Cc-ME-mediated suppression of NO production is not caused by a cytotoxic effect but stems from its specific anti-inflammatory activity in macrophages. Therefore, we next attempted to identify the components in Cc-ME that generate an anti-inflammatory effect and suppress the production of NO in macrophages. To that end, we performed HPLC analysis of the standard anti-inflammatory flavonoids quercetin, luteolin, and kaempferol [37, 38]. The HPLC analysis showed that Cc-ME contained all three anti-inflammatory flavonoids, with luteolin content being the highest (Figure 1(d)), suggesting that the luteolin in Cc-ME might be the main component of its anti-inflammatory activity. We tested this possibility and found that luteolin significantly inhibited NO production in macrophages in a dose-dependent manner (data not shown), as reported previously [39]. Thus, we found strong indications that luteolin is the main phytochemical compound in Cc-ME that provides an anti-inflammatory effect.

Because NO production is catalyzed by the enzyme iNOS, we investigated the effect of Cc-ME on mRNA expression of iNOS in LPS-stimulated macrophages. As expected, semiquantitative and quantitative RT-PCR results clearly showed that Cc-ME suppressed mRNA and protein expression of iNOS in LPS-treated macrophages in a dose-dependent manner (Figures 2(a), 2(b), and 2(c)). The effect of Cc-ME on the mRNA expression of other inflammatory genes was also examined in LPS-stimulated macrophages, and Cc-ME effectively suppressed mRNA expression of COX-2, IL-1*β*, IL-6, and TNF-*α* from both RAW264.7 cells and peritoneal macrophages (Figure 2). In addition, anti-inflammatory cytokines such as IL-10 and TGF-*β* were found to be increased by Cc-ME, implying that the balance of inflammation and anti-inflammatory conditions are critically regulated by this extract. Because mRNA expression of iNOS, COX-2, and TNF-*α* is commonly induced by the inflammatory transcription factor NF-*κ*B, we examined the effect of Cc-ME on NF-*κ*B activity by determining nuclear translocation of NF-*κ*B in LPS-stimulated macrophages [42]. The results showed that nuclear translocation of both p65 and p50, two components of NF-*κ*B, was inhibited by Cc-ME in LPS-stimulated macrophages (Figure 2(d)), implying that Cc-ME downregulates mRNA expression of inflammatory genes, including iNOS, COX-2, and TNF-*α* by inhibiting nuclear translocation and activity of NF-*κ*B in macrophages during inflammatory responses.

Because Cc-ME effectively suppressed the nuclear translocation of NF-*κ*B in LPS-treated macrophages, we evaluated the effect of Cc-ME on the activity of upstream signaling molecules such as Src, PDK1, AKT, and IKK in the NF-*κ*B pathway of LPS-stimulated macrophages. Interestingly, Cc-ME suppressed the kinase activities of Src, p85, AKT, IKK*α*/*β*, and I*κ*B*α* (Figures 3(a) and 3(b)). Src and p85 are the most upstream kinases in NF-*κ*B signaling pathway [4]. Therefore, we expected Src and p85 to be activated rapidly with LPS stimulation and evaluated the effect of Cc-ME on their activity at early time points. In accordance with Figure 3(a), Cc-ME effectively suppressed the activity of Src and p85 in LPS-stimulated macrophages at 2, 3, and 5 min (Figure 3(c)), indicating that Cc-ME targets very upstream signaling molecules in its anti-inflammatory activity. This idea was confirmed using Src-overexpressed HEK293 cells. As expected, Cc-ME dramatically suppressed the activity of Src as well as the activity of downstream signaling molecules of Src in the NF-*κ*B pathway in the Src-transfected HEK293 cells (Figure 3(d)), strongly indicating that Src is a direct target of Cc-ME's anti-inflammatory activity in the NF-*κ*B signaling pathway during macrophage-mediated inflammatory responses. Because inflammatory signal cascades are transduced by a series of phosphorylation chain reactions of intracellular kinases, we suggest that Cc-ME might interrupt the interactions among those kinases. Because Cc-ME suppressed the most upstream signaling molecule, Src, we evaluated the effect of Cc-ME on the interaction between Src and p85, a downstream signaling molecule of Src, in LPS-stimulated macrophages. As expected, Cc-ME effectively inhibited the molecular interaction between Src and p85 induced by LPS in macrophages (Figure 3(e)). These results indicate that Cc-ME suppresses the inflammatory NF-*κ*B signaling pathway by targeting Src and interfering with the necessary molecular interaction between Src and p85. Similar to our findings, numerous amounts of evidence have also supported the significance of Src in NF-*κ*B activation during inflammatory and cancerous reactions [43, 44]. Thus, chemical suppression of Src by PP2 reduced the activation of NF-*κ*B linked to suppression of inflammatory responses [45]. Newly identified inhibitors to Src have been also proved as anti-inflammatory and anti-cancer drug candidates [43, 46]. Furthermore, biochemical analysis on the substrates of Src revealed that a regulatory subunit (p85) of phosphatidylinositol-4,5-bisphosphate 3-kinase (PI3K) is phosphorylated and therefore PI3K-mediated signaling cascades composed of phosphoinositide-dependent kinase-1 (PDK1), AKT, and IKK play a critical role in the activation of NF-*κ*B [47, 48]. Based on our results and previous reports, it is conclusive that Cc-ME-mediated Src suppression might maintain strong suppressive conditions of TLR4-induced inflammatory responses by reduction of Src-processed downstream signaling cascades essential for NF-*κ*B activation. In spite of this possibility, however, we will also continue to test whether Cc-ME is able to suppress the upstream event of TLR4 activation such as dimerization of TLR4, since suppression of this pathway can also block the activation of upstream kinase Src or Syk. For this, we will perform immunoprecipitation analysis to check molecular interaction pattern with TLR4 containing different tagging proteins.

We further investigated* in vivo* anti-inflammatory effect of Cc-ME in mouse models of two acute inflammatory diseases: LPS-induced peritonitis and HCl/EtOH-induced gastritis. Because peritonitis causes the recruitment of leukocytes, especially inflammatory innate cells in the peritoneal cavity of diseased mice, we evaluated an anti-inflammatory activity of Cc-ME by determining the total number of leukocytes in the cavity of peritonitis mice. Cc-ME dose-dependently inhibited the recruitment of leukocytes in the cavity of peritonitis mice, and its effect at 200 mg/kg was comparable with that of prednisolone, which is the anti-inflammatory drug approved to treat that condition (Figure 4(a)). By flowcytometric analysis, indeed, it was found that the cell population of macrophages but not B and T cells was significantly reduced by Cc-ME (Figures 4(b) and 4(c)), implying that macrophage migration can be interfered by this extract, in addition to* in vitro* suppressive activity of Cc-ME on macrophage activation during LPS exposure (Figures 1 and 2). Cc-ME also exerted an anti-inflammatory activity in a gastritis mouse model by dramatically ameliorating the ulcerative lesions in the stomachs of gastritis mice and by decreasing the mRNA expression of inflammatory genes (Figures 4(b) and 4(c)). It is reasonable to think that this* in vivo* anti-inflammatory activity of Cc-ME is mediated by the luteolin, quercetin, and kaempferol contained in Cc-ME because those compounds have already been observed to show anti-inflammatory effects that ameliorate the morphological finding of peritonitis and gastritis [33, 49–53]. Taken together, these results strongly indicate that Cc-ME has an* in vivo* anti-inflammatory activity that can ameliorate the morphological finding of inflammatory diseases and also suggest that the flavonoids in Cc-ME might be the main components of its* in vivo* anti-inflammatory effect.

**Figure 5:**
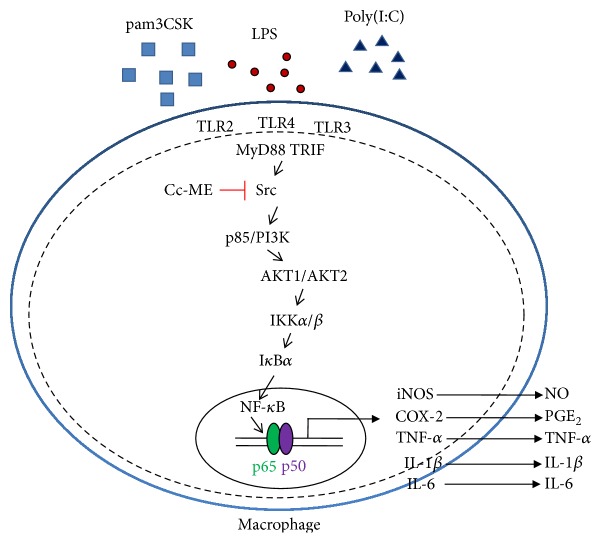
Proposed model for Cc-ME-mediated anti-inflammatory activity by targeting Src in NF-*κ*B signaling pathway during macrophage-mediated inflammatory responses.

In conclusion, we investigated* in vitro* and* in vivo* anti-inflammatory activities of Cc-ME in macrophages and acute inflammatory disease models in mice and successfully demonstrated that Cc-ME shows a strong anti-inflammatory activity by targeting Src in the NF-*κ*B signaling pathway during macrophage-mediated inflammatory responses (Figure 5). Since T cells are regarded as important cells in chronic inflammatory diseases, we will also continue to examine whether Cc-ME can also interrupt the functional role of T cells in various chronic inflammatory and autoimmune diseases such as collagen-induced arthritis. Taken together, our findings might promote understanding of the anti-inflammatory activity of* Celtis choseniana* on a molecular level and provide a possibility of developing a potential anti-inflammatory remedy which can be used to prevent and treat various human inflammatory diseases.

## Figures and Tables

**Table 1:** Instrument and working conditions for quercetin, luteolin, and kaempferol analysis by high-performance liquid chromatography (HPLC).

Instrument	KNAUER Corp. HPLC system	
Column	Phenomenex, Gemini 5 *μ* C18 110A, 250 × 4.60 mm 5 *μ*m	
Detector	UV/VIS detector (370 nm)	
Solvent A	0.1% TFA in H_2_O	
Solvent B	0.08% TFA in 95% MeCN + 5% H_2_O	
Standard	Dilution with DMSO	
Sample treatment	50 mg/mL dilution with DMSO	
Injection volume	20 *μ*L	
Flow rate	1.0 mL/min	
Gradient elution system	% A	% B
Time (min)		
0	100	0
50	50	50
60	0	100

**Table 2:** PCR primers sequence used in this study.

Name		Sequence (5′ to 3′)
*Semiquantitative PCR*		
iNOS	Forward	CCCTTCCGAAGTTTCTGGCAGCAG
	Reverse	GGCTGTCAGAGCCTCGTGGCTTTGG
COX-2	Forward	CACTACATCCTGACCCACTT
	Reverse	ATGCTCCTGCTTGAGTATGT
TNF-*α*	Forward	TTGACCTCAGCGCTGAGTTG
	Reverse	CCTGTAGCCCACGTCGTAGC
GAPDH	Forward	CACTCACGGCAAATTCAACGGCA
	Reverse	GACTCCACGACATACTCAGCAC

*Real-time PCR*		
iNOS	Forward	GGAGCCTTTAGACCTCAACAGA
	Reverse	TGAACGAGGAGGGTGGTG
COX-2	Forward	CACTACATCCTGACCCACTT
	Reverse	ATGCTCCTGCTTGAGTATGT
TNF-*α*	Forward	GCCTCTTCTCATTCCTGCTTG
	Reverse	CTGATGAGAGGGAGGCCATT
IFN-*β*	Forward	AAGAGTTACACTGCCTTTGCCATC
	Reverse	CACTGTCTGCTGGTGGAGTTCATC
IFN-*γ*	Forward	GGGTTGTTGACCTCAAACTTGGCA
	Reverse	CAGGCCATCAGCAACAACAT
IL-1*β*	Forward	CAACCAACAAGTGATATTCTCCATG
	Reverse	GATCCACACACTCCAGCTGCA
IL-6	Forward	CTAGGTTTGCCGAGTAGATCTC
	Reverse	GACAAAGCCAGAGTCCTTCAGAGA
IL-10	Forward	ATAACTGCACCCACTTCCCA
	Reverse	GGGCATCACTTCTACCAGGT
TGF-*β*	Forward	AACAATTCCTGGCGTTACCTT
	Reverse	CTGCCGTACAACTCCAGTGA
GAPDH	Forward	CAATGAATACGGCTACAGCAAC
	Reverse	AGGGAGATGCTCAGTGTTGG

COX-2, cyclooxygenase-2; GAPDH, glyceraldehyde 3-phosphate dehydrogenase; iNOS, inducible nitric oxide synthase; IFN-*β*, interferon-beta; IFN-*γ*, interferon-gamma; IL-1*β*, interleukin-1 beta; IL-6, interleukin 6; PCR, polymerase chain reaction; TNF-*α*, tumor necrosis factor-alpha.

## Abbreviations

LPS: Lipopolysaccharide
pam3: pam3CSK4
L-NAME: N(G)-Nitro-L-arginine methyl ester
NO: Nitric oxide
iNOS: Inducible nitric oxide synthase
COX: Cyclooxygenase
TNF-*α*: Tumor necrosis factor-alpha
GAPDH: Glyceraldehyde 3-phosphate dehydrogenase
TLR: Toll-like receptor
RT-PCR: Reverse transcriptase-polymerase chain reaction
NF-*κ*B: Nuclear factor-kappa B
I*κ*B*α*: Inhibitor of kappa B alpha
IKK: I*κ*B kinase.
